# Microsomic and macrosomic body structure in children and adolescents affected by syndromes or diseases associated with neurodysfunction

**DOI:** 10.1038/s41598-021-85587-7

**Published:** 2021-03-18

**Authors:** Lidia Perenc, Agnieszka Guzik, Justyna Podgórska-Bednarz, Mariusz Drużbicki

**Affiliations:** Department of Physiotherapy, Institute of Health Sciences, College of Medical Sciences, University of Rzeszów, Rzeszów, Poland

**Keywords:** Neurodevelopmental disorders, Paediatric neurological disorders

## Abstract

In Poland the issue of microsomic body structure (micro-SBS) and macrosomic body structure (macro-SBS) has so far been overlooked. Up until now only a small amount of data have been published, most often as an overview of the problem. The current study was designed to investigate the co-occurrence of microsomic/macrosomic body structure (micro/macro-SBS) and congenital nervous system disorders or neurological syndromes with symptoms visible from infancy, based on essential data acquired during admission procedures at a neurological rehabilitation ward for children and adolescents. The study applied a retrospective analysis of data collected during hospitalization of 327 children and adolescents, aged 4–18 years who had been affected since infancy by congenital disorders of the nervous system and/or neurological syndromes associated with a minimum of one neurodysfunction. To identify subjects with microsomic or macrosomic body structure in the group of children and adolescents, the adopted criteria took into account z-score values for body height (z-score Ht), body weight (z-score Wt), head circumference (z-score HC), BMI (z-score BMI) and head circumference index (z-score HCI). The rates of micro/macro-SBS in the study group amounted to 7.3% and 0.6%, respectively. The findings show a more frequent co-occurrence of, as well as statistically significant correlations between, micro/macro-SBS and type of spasticity (cerebral palsy) (p = 0.024) as well as hydrocephalus not treated surgically (p < 0.001). Macro-SBS was found to more frequently co-occur with hemiplegia and hydrocephalus not treated surgically.

## Introduction

In the related literature we can encounter such terms as ‘small for gestational age’, ‘normal for gestational age’ or ‘large for gestational age’^1–3^. ‘Small for gestational age/large for gestational age’ may be defined as insufficient/excessive body weight at birth relative to gestational age^1^. Estimated fetal weight is calculated based on antenatal ultrasonographic measurements performed while assessing fetal biparietal diameter (cm), head circumference (cm), abdominal circumference (cm) and femur length (cm), with the use of a Hadlock algorithm^3^. Impaired fetal growth, insufficient for gestational age, also referred to as intrauterine hypotrophy, is a consequence of intrauterine or fetal growth restriction^2,3^. Notably, children with intrauterine hypotrophy do not constitute a uniform group. Some of them are found with symmetric hypotrophy, i.e. body weight small for gestational age, as well as deficient dimensions of long bones, and body circumferences. Others present asymmetric hypotrophy, i.e. body weight small for gestational age and the remaining measures more or less corresponding to the gestational age^2^. The terms ‘large for gestational age’ and ‘fetal macrosomia’ are sometimes used interchangeably^3,4^.

It seems that as many as one in twenty neonates may be affected by hypotrophy^5^, while increased fetal weight is estimated to occur in approximately 10% of all pregnancies^6,7^. A growing body of evidence reported in recent years shows associations between intrauterine growth and the person’s health status later in life. Intrauterine hypotrophy/hypertrophy of the fetus is a consequence of a wide range of pathological processes in various periods of gestation^5^.

Symmetric hypotrophy of the fetus is a consequence of chromosomal aberrations, monogenic disorders of the fetus, congenital metabolic disorders of the fetus, intrauterine infections and exposure to harmful chemical factors (e.g. alcohol)^2^. The most common causes of asymmetric hypotrophy include placental abnormalities, such as premature aging or partial detachment, as well as thrombosis of placental vessels or maternal diseases (hypertension, anemia, malnutrition, diabetes, heart and kidney diseases and nicotinism)^2,5,8^. Birth weight classified as small for gestational age more commonly occurs in children born from multiple pregnancies or those born prematurely. Additionally, those born pre-term not only present complicated problems related to postnatal adaptation in infancy, but are also affected by many disorders occurring later in life, e.g. growth defects, neurological and intellectual consequences, metabolic syndrome, as well as pulmonary and cardio-vascular complications and impaired kidney function^5,8^.

It is assumed that fetal macrosomia is usually induced by maternal factors: age over 35 years, body type, multiple births, gestational diabetes, high arterial pressure during pregnancy, delayed birth, obesity during pregnancy or large increase in body weight during pregnancy^9–11^. On the other hand, the fetal risk factors for macrosomia include genetically determined congenital malformations. It has also been pointed out that fetal macrosomia may be linked to the procedure of embryo cryopreservation during in vitro fertilization^4^. In the case of macrosomia, natural childbirth increases the risk of severe complications in the mother and is associated with greater risk of perinatal injury of the fetus. Perinatal complications may contribute to the development of encephalopathy, resulting, for example, in intellectual disability^9–11^.

In the period of intranatal and perinatal development, the occurrence of children with symmetric and asymmetric hypotrophy and macrosomia is observed^1–11^. The environment in the uterus and in the early stages of a newborn's life can provoke a sustained response in the fetus and the newborn. For example, fetal hypertrophy is associated with exposure to a diabetic intrauterine environment, which increases the susceptibility to intergenerational obesity. Fetal growth restriction is complex as it may be affected by malnutrition in the uterus, catching up in growth due to high caloric intake and low levels of physical activity later in life^12^. Although, in small for gestational age neonates postnatal catch-up growth (defined as the difference in both body height and body weight between 4 weeks and 3 years of age) is similar to appropriate for gestational age neonates, small for gestational age neonates tend to have a significantly lower body height and weight compared to appropriate for gestational neonates for all available measurement moments up to 3 years of age^13^.

In view of the above, it may be hypothesized that a population of children and adolescents will comprise some individuals with body measures (body weight, body height, head circumference) below and above the norm. The authors understand microsomic body structure (micro-SBS) as a complex of developmental disorders characterized by the coexistence of low body weight, short stature and microcephaly. They treat macrosomic body structure (macro-SBS) as an opposing complex of developmental disorders characterized by high body weight, tall stature and macrocephaly. Micro-SBS is a concept akin to hypotrophy/small for gestational age and macro-SBS is akin to macrosomia/large for gestational age—but they are not the same. During ontogenetic development, the process of differentiation of body proportions takes place parallel to the growth process—with age, body dimensions increase and body proportions change^14,15^. The question was whether the body structure in case of micro-SBS and macro-SBS is proportional. Taking into account that the literature on the subject contains descriptions of the occurrence of disorders of somatic development (e.g. short stature)^16,17^ and neurodevelopmental disorders^16^ in children and adolescents with congenital nervous system disorders^17,18^ or neurological syndromes with symptoms visible from infancy^16^, research was conducted in this group of children and adolescents.

Disorders of the growth process^19^ and differentiation of body proportions^20^ can be determined on the basis of physical examination^19–21^. As is commonly known, diagnosing diseases with damage to the nervous system in children and adolescents is a difficult task^22–24^. Only an appropriate clinical deduction and a properly planned diagnostic and differentiating process can ensure success and correct diagnosis^25^. However, our observations show that a significant number of children and adolescents with congenital disorders of the nervous system or neurological syndromes with one or more neurodysfunctions visible from infancy have not been diagnosed correctly.

Based on a review of the literature, it can be concluded that in Poland no studies so far have investigated the co-occurrence of micro-SBS or macro-SBS and congenital disorders of the nervous system or neurological syndromes with symptoms visible from infancy in children and adolescents. Therefore, this article is the first one to present scientific evidence related to this issue in Poland.

The purpose of the current study was to investigate the co-occurrence of micro/macro-SBS and congenital nervous system disorders or neurological syndromes with symptoms visible from infancy, based on essential data acquired during admission procedures at a neurological rehabilitation ward for children and adolescents. The co-occurrence of micro-SBS and macro-SBS with individual diagnoses and separate subgroups in this group of children and adolescents was analyzed. It was also noted in which subgroups micro/macro-SBS does not exist.

## Material and methods

The methodology used in this study is consistent with previous studies on the coexistence of short-stature^19^, tall-stature^19^, relative/absolute microcephaly^20^ and relative/absolute macrocephaly^20^ and congenital nervous system disorders or neurological syndromes with symptoms visible from infancy, based on essential data acquired during admission procedures at a neurological rehabilitation ward for children and adolescents. The same criteria for inclusion and exclusion from the study were used (the study group was the same), anthropometric parameters were used (in this study, body weight and body mass index were additionally used), as well as the Gross Motor Function Classification System (GMFCS)^19,20^. This publication presents the criteria for the diagnosis of disorders of body structure: micro/macro-SBS and, using analogous, selected statistical methods, the occurrence of the disorders of body structure in the study group was analyzed (the occurrence of the disorders of body structure’ it seems very unlikely that it has not been discussed before).

### Participants

**Figure 1:**
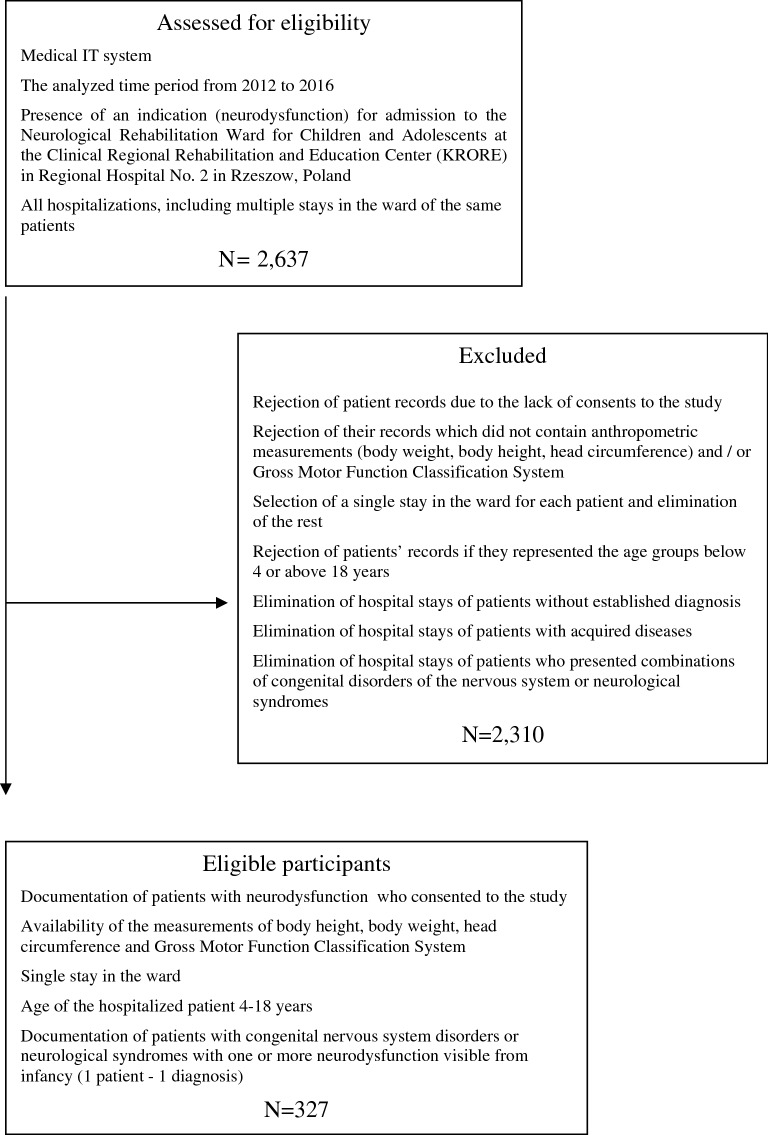
Flow of subjects through the study.

The retrospective study took into account information related to 327 children and adolescents admitted between 2012 and 2016 to the Neurological Rehabilitation Ward for Children and Adolescents in Regional Hospital No. 2 in Rzeszow, Poland, and staying at the Clinical Regional Rehabilitation and Education Centre (KRORE). The group of patients was deliberately selected, because researchers were interested in a specific group of diseases/syndromes (Fig. 1). All the patients eligible for the study were hospitalized in the period from 2012 to 2016 and presented congenital nervous system disorders or neurological syndromes with one or more neurodysfunctions visible from infancy. It was assumed, according to other authors, that neurodysfunctions are varied symptoms resulting from damage to different parts of the nervous system or motor unit, e.g. developmental delay, altered gait, cranial nerve palsies, tremors, paralysis, urinary incontinence^26–28^.

The following additional eligibility criteria were adopted: age of 4–18 years, informed consent from both the children and their parents/legal guardians, availability of the measurements of body height, body weight, head circumference and GMFCS, as well as complete diagnostic data, all of which were acquired during a single admission procedure^19,20^.

Patients were excluded from the study if they had no diagnosis of a congenital disorder of the nervous system or a neurological syndrome linked with one or more neurodysfunction visible from infancy, were diagnosed with an acquired disease or if they presented combinations of congenital disorders of the nervous system or neurological syndromes (e.g. Down’s syndrome, neural tube defect or phenylketonuria co-occurring with cerebral palsy). Additionally, patients were excluded if they were not hospitalized in the relevant period, if there was more than one admission procedure, if they represented the age groups below 4 or above 18 years (due to the lack of biological frame of reference), if their records did not contain complete diagnostic information and/or anthropometric measurements (body weight, body height, head circumference) and GMFCS, and finally if no informed consent was given by the children and their parents/legal guardians^19,20^.

A total of 2,637 hospitalizations took place from 2012 to 2016 in the Neurological Rehabilitation Ward for Children and Adolescents at the Regional Hospital No. 2 in Rzeszow, Poland of patients who stayed at KRORE. Of these, 327 patients were found to meet the inclusion criteria; as a result the retrospective analysis took into account 327 children and adolescents (143 girls—43.7%, 184 boys—56.3%), with a mean age of 9.7 ± 4.3 years (median 9.0 years; the youngest child was 4 and the oldest was 18 years of age)^19,20^.

The study protocol was accepted by the Bioethics Commission at the University of Rzeszow, Poland, and the procedures used complied with the applicable guidelines and regulations. Before the application was filed with the bioethics committee, informed consents were obtained from the patients and their parents/legal guardians as well as the director of the hospital.

### Procedures and data analyses

The basic data taken into account in the retrospective analysis included the patients’ age, sex, as well as principal and additional diagnosis, body weight—Wt, body height—Ht, head circumference—HC. All the information was retrieved from the patient records collected at admission. The relevant anthropometric measurements had been carried out by the hospital personnel in compliance with the guidelines approved at KRORE. The diagnoses, both principal and additional, had been specified by neurologists, geneticists, endocrinologists and other specialists before admission to KRORE. According to the medical records, the children presented a variety of conditions or syndromes associated with damage to the nervous system. All of these were congenital anomalies and/or disorders, with or without encephalopathy, and accompanied by motor defects (neurodysfunctions) visible from early childhood. The criteria reported in the related literature (i.e. suspected encephalopathy or no encephalopathy, its etiopathogenesis and nature)^21^ were applied in dividing the patients into subgroups (Table 1A). Given the diverse nature of neural tube defects^29^ and the critical importance of the operative treatment for the person’s development in the future^30^, the neural tube defects subgroup was further divided to take into account the cause of the surgery, i.e. myelomeningocele with hydrocephalus (sasMMC&HCP) and myelomeningocele alone (sasMMC). The analysis also includes the cases where no surgery was performed. Chromosomal aberrations and genetic mutations were included in the subgroup with genetic disorders, as in other studies^21,22^. Based on the diagnostic criteria proposed by Hagberg^11^ it the types of cerebral palsy were determined.

**Table 1:** The diagnostic criteria for micro/macro-SBS (A), the level of GMFCS (B), the division of group, the numbers in subgroups (C), the statistical characteristics of z-scores: Ht, Wt, HC, BMI, HCI (D), the numbers in micro-SBS and macro-SBS in the study group (E), the statistical characteristics of the GMFCS score in the study group (F).

A. The division of the group and the numbers in subgroups								
Classification with regard to etiopathogenesis, presence and character of encephalopathy			Classification with regard to presence and character of encephalopathy					
	N	%		N	%			
MD—metabolic disorder	7	2.1	PE—progressive encephalopathy	8	2.4			
EE—epileptic encephalopathy	1	0.3						
NTDs—neural tube defects	24	7.3	NPE—non-progressive encephalopathy	287	87.7			
GD—genetic disorders	23	7.0						
TE—toxic encephalopathy	1	0.3						
CP—cerebral palsy	239	73.1						
NMD—neuromuscular disorders	32	9.8	NMD—neuromuscular disorders	32	9.8			
*N* numbers of patients, *%* percent								

B. The diagnostic criteria for micro-SBS (proportional and disproportional) and macro-SBS (proportional and disproportional)								
Body structure	z-score Ht	z-score Wt	z-score HC	z-score BMI	z-score HCI			
Micro-SBS Proportional	z-score h < − 2	z-score w < − 2	z-score hc < − 2	− 1 ≥ z-score BMI ≤ 1	− 2 ≥ z-score HCI ≤ 2			
Micro-SBS Disproportional	z-score h < − 2	z-score w < − 2	z-score hc < − 2	− 1 < z-score BMI > 1	− 2 < z-score HCI > 2			
Macro-SBS Proportional	z-score h > 2	z-score w > 2	z-score hc > 2	− 1 ≥ z-score BMI ≤ 1	− 2 ≥ z-score HCI ≤ 2			
Macro-SBS Disproportional	z-score h > 2	z-score w > 2	z-score hc > 2	− 1 < z-score BMI > 1	− 2 < z-score HCI > 2			
*micro-SBS* microsomic body structure, *macro-SBS* macrosomic body structure								

C. The level of GMFCS—the higher the number of points, the greater the level of motor disability								
GMFCS I–V	I	II	III	IV	V			
number of points assigned	1	2	3	4	5			
GMFCS A–C	A		B	C				
number of points assigned	1		2	3				
GMFCS—Gross Motor Function Classification System								

D. The statistical characteristics of z-scores: Ht, Wt, HC, BMI, HCI								
z-score	N	$\overline{x}$	Me	*s*	*c*_25_	*c*_75_	Min	Max
z-score Ht	327	− 1.23	− 1.16	1.98	− 2.33	− 0.05	− 8.93	4.20
z-score Wt		− 0.78	− 1.05	1.98	− 1.90	0.17	− 10.52	8.75
z-score HC		− 0.53	− 0.54	2.14	− 1.71	0.86	− 7.36	8.29
z-score BMI		− 0.33	− 0.64	1.76	− 1.45	0.6	− 4.20	6.98
z-score HCI		0.90	0.85	2.04	− 0.44	2.20	− 4.8	11.29
*Ht* height, *Wt* weight, *HC* Head Circumference, *BMI* body mass index, *HCI* head circumference index, *N* numbers of patients, $\overline{x}$ arithmetic mean, *Me* median, *s* standard deviation, *Min* smallest value, *Max* largest value, *c*_*25*_ 25th centile and *c*_*75*_ 75th centile								

E. The numbers in micro-SBS and macro-SBS in the study group								
Developmental disorders	N		%					
Micro-SBS	24		7.3					
Macro-SBS	2		0.6					
Overall	26		7.9					
No disorders	301		92.1					
In total	327		100.0					
*micro-SBS* microsomic body structure, *macro-SBS* macrosomic body structure, *N* numbers of patients, *%* percent								

F. The statistical characteristics of the GMFCS score in the study group								
Parameter	Study group	N	$\overline{x}$	Me	*s*	Min	Max	
GMFCS I–V	Entire study group	327	2.48	2.0	1.30	1	5	
GMFCS A–C	Entire study group	327	1.60	1.0	0.86	1	3	
GMFCS I–V	Micro-SBS	24	3.29	3.5	1.55	1	5	
GMFCS A–C	Micro-SBS	24	2.12	2.5	0.95	1	3	
GMFCS I–V	Macro-SBS	2	1.50	1.5	0.71	1	2	
GMFCS A–C	Macro-SBS	2	1.00	1.0	0.00	1	1	
*GMFCS* Gross Motor Function Classification System, $\overline{x}$ arithmetic mean, *Me* median, *s* standard deviation, *Min* smallest value, *Max* largest value, *c*_*25*_ 25th centile and *c*_*75*_ 75th centile								

The ratio of Body Mass Index (BMI) to Head Circumference Index (HCI), i.e. the quotient of head circumference and body height (HC/Ht), was calculated for each patient. In order to assess development deficits based on all of the previously mentioned parameters, z-scores were calculated for head circumference (z-score HC), body height (z-score Ht), body weight (z-score Wt), body mass index (z-score BMI) and head circumference index (z-score HCI). Normative values published earlier were applied as a reference^14,15^.

In order to identify those children and adolescents in the study group who had micro-SBS and macro-SBS, it was determined that the adopted criteria (Table 1B) should take into account z-score values for body height (z-score Ht), body weight (z-score Wt), head circumference (z-score HC), BMI (z-score BMI) and head circumference index (z-score HCI). The criteria were established based on the differences between normal and abnormal development, taking into account the process of growth and differentiation of the proportions of the body^31–44^. Consequently, the following terms were introduced: proportional micro-SBS, disproportional micro-SBS, proportional macro-SBS and disproportional macro-SBS (Table 1B).

When developing the above criteria for identifying developmental disorders of body structure, the authors were guided by the most frequently used limits of developmental norms. As is known, anthropometric measurements are always assessed taking into account age and gender, in relation to percentile grids or tables of norms. 68.26% of the anthropometric measurements are included within the interval of one standard deviation above and below the mean—these are always correct values. The most common limit values are: 3rd percentile (− 1.88 standard deviations) and 97th percentile (+ 1.88 standard deviations) or 10th percentile (− 1.28 standard deviations) and 90th percentile (+ 1.28 standard deviations). Values below the 3rd percentile (− 1.88 standard deviations)/10th percentile (− 1.28 standard deviations) are too small for age and gender and above the 97th percentile (+ 1.88 standard deviations)/90th percentile (+ 1.28 standard deviations) are too large for age and gender^33^.

Growth disturbances were considered (physiologically, with age the proportions of the body change). The authors assumed that the micro-SBS complex of developmental disorders are characterized by the coexistence of low body weight, short stature and microcephaly. For low body weight the authors assumed Wt more than 2 standard deviations below the mean for age (z-score Wt < − 2). The authors also adopted a similar criterion when assessing malnutrition^34^. In the subject literature, short stature is defined as a Ht more than 2 standard deviations below the mean for age (less than the 3rd percentile)^31,32, 35^. For short stature the authors assumed a z-score Ht < -2. The criterion used to diagnose microcephaly is HC more than 2 standard deviations below the mean for age^36^. Similarly, for microcephaly, the authors adopted a z-score HC < 2. Macro-SBS as an opposing complex of developmental disorders is characterized by high body weight, tall stature and macrocephaly. As a criterion for high body weight (z-score Wt > 2), the criterion for the diagnosis of macrosomia was adopted: Wt more than 2 standard deviations above the mean for age^37^. Tall stature is commonly defined as a height more than 2 standard deviations above the mean for age (greater than the 97th percentile)^35^. As a criterion for tall stature a z-score Ht > 2 was established, and for macrocephaly: a z-score HC > 2—a criterion also used by other authors (diagnosis of macrocephaly when HC is more than 2 standard deviations above the mean for age)^38^. Disturbances in differentiation of body proportions were considered (physiologically, with age the proportions of the body change). HCI (ratio of head circumference to body height) reflects the differentiation of head size in relation to body height. Normal values are in the range: − 2 ≥ z-score HCI ≤ 2. A z-score HCI < − 2 tells us that the head is too small in relation to the height of the body, z-score HCI > 2 tells us that the head is too big for body height.

The differentiation of the weight-to-height ratio is reflected by the BMI. Based on the BMI value, the nutritional status is assessed. The division of eating disorders is defined differently by different authors^39–41^. Malnutrition was defined as a BMI value, age- and sex-specific, under the 15th percentile^39^. According to the definitions developed by the Center for Disease Control and Prevention, overweight is defined as a BMI at or above the 85th percentile and lower than the 95th percentile, and obesity—a BMI at or above the 95th percentile^40^. As can be seen, the limits of the norm are narrower (16–84th percentile)—which is different than in the previous cases. Therefore, for the correct differentiation of body proportions, the range − 1 ≥ z-score BMI ≤ 1 was adopted. Children with such BMI values have normal weight-to-height proportions, which excludes the occurrence of the above-mentioned nutritional status disorders. The diagnosis of a nutritional disorder: malnutrition^39,42,43^, overweight^40,44^, obesity^40,44^ obliges diagnostic and therapeutic actions to be carried out^40,42,43,45–47^. If there are no disturbances in differentiating body proportions, micro/macro-SBS is defined as proportional, and if there is at least one of the abnormalities of differentiation of body proportions described above, it is disproportional.

Assessment of the severity of disability in the entire study group is based on the five-step Gross Motor Function Classification System—GMFCS I-V^48^. In order to be statistically significant, levels I and II of GMFCS have been combined as group A, levels IV and V—as group C, and level III corresponds to group B—GMFCS A-C^20^ (Table 1C). In Poland, each patient with neurodysfunction (with and without CP) admitted to the Neurological Rehabilitation Ward for Children and Adolescents is assessed on the GMFCS scale^49,50^.

The analyses were designed to identify any correlations of co-existing development defects, in particular micro/macro-SBS and disorders or syndromes associated with neurodysfunction, as well as the subgroups distinguished. Additional diagnoses were also taken into account. Adjusted Standardized Residuals (ASR) were computed, similar to the analyses presented earlier^19,20^. Values higher than 1.96 correspond to a higher number, and those lower than − 1.96 represent a lower number than a random distribution. The Pearson chi-square test only tells us if a relationship exists (e.g. there is a relationship between micro/macro-SBS and hemiplegia). ASR is valuable as it provides additional information about the type of this relationship (e.g. macro-SBS relatively more often coexists with hemiplegia and micro-SBS relatively less frequently)^51^. Statistical inference methods were used to determine in what way the intergroup differences reflect certain regularities in the relevant population, or whether they are random. Due to the nominal nature of the characteristics being compared, a chi-square test of independence was further applied. Nominal regression was used to assess the relationships between the dependent qualitative and independent quantitative variables. A value of p < 0.05 was assumed to reflect statistical significance. Pearson’s Contingency Coefficient C (Cp) can only take positive values (Cp ≥ 0). A relationship is reflected by a Cp distant from 0, while values approaching 1 show a near-perfect association. Comparisons in two groups of quantitative variables were made with the Mann Whitney U test.

## Results

The structure of the study group is presented below again^19,20^. Out of the seven subgroups that were distinguished in the study group, six comprised patients with medical conditions commonly associated with encephalopathy: progressive metabolic disorders (2.1%), progressive genetically-determined epileptic syndromes (0.3%), non-progressive neural tube defects (7.3%), non-progressive genetic disorders: chromosomal aberrations, monogenic disorders except neuromuscular diseases (7.0%), non-progressive toxic encephalopathy (0.3%) and non-progressive cerebral palsy (73.1%). One more subgroup comprised children with conditions that are not generally associated with encephalopathy, i.e. neuromuscular diseases (9.8%). The nature and expected presence of encephalopathy^13^ were used as criteria according to which these six subgroups were combined into two large groups representing conditions associated with progressive encephalopathy (2.4%) and non-progressive encephalopathy (87.7%). The third group comprised children with neuromuscular diseases (9.8%) (Table 1A).

Progressive metabolic disorders were represented by: 2 persons (0.6%) with neurodegeneration due to brain iron accumulation—mitochondrial protein associated neurodegeneration and 1 (0.3%) person each representing the following diagnoses: Pompe disease, long-chain 3-hydroxyacyl-coenzyme A dehydrogenase deficiency, Smith-Lemli-Opitz syndrome, glucose transporter type 1 deficiency, nonketotic hyperglycinemia. There was also 1 person with Dravet syndrome (0.3%) in the progressive genetically-determined epileptic syndromes subgroup. In the neural tube defects subgroup there occurred: states following surgery due to lumbar myelomeningocele with hydrocephalus (N = 17, 5.2%), states following surgery due to lumbar myelomeningocele (N = 3, 0.9%), state following surgery due to parieto-occipital meningocele (N = 1, 0.3%), Arnold-Chiari malformation (N = 2, 0.6%) and isolated hydrocephalus (N = 1, 0.3%). The genetic disorders subgroup was the most diverse: Down’s syndrome (N = 11, 3.4%), Edwards syndrome (N = 1, 0.3%), Phelan-McDermid syndrome (N = 2, 0.3%), Mowat-Wilson syndrome (N = 1, 0.3%), Angelman syndrome (N = 1, 0.3%), DiGeorge syndrome (N = 1, 0.3%), 46,XY,del(X)(q24) (N = 1, 0.3%), Cornelia de Lange syndrome (N = 1, 0.3%), Shwachman-Diamond syndrome (N = 1, 0.3%), Prader-Willi syndrome (N = 1, 0.3%), 46 XX, add(2)(q25) (N = 1, 0.3%), 46XX, del (12) (q24.21q24.23) (N = 1, 0.3%). Fetal alcohol syndrome (N = 1, 0.3%) represented the non-progressive toxic encephalopathy subgroup. Cerebral palsy was the most numerous subgroup (N = 239, 73.1%). In the neuromuscular diseases subgroup there occurred: hereditary motor and sensory polyneuropathy (N = 8, 2.4%), limb-girdle muscular dystrophy (N = 7, 2.1%), Becker muscular dystrophy (N = 3, 0.9%), Duchenne muscular dystrophy (N = 7, 2.1%), Thomsen disease (N = 1, 0.3%), arthrogryposis multiplex congenita with neuropathy (N = 3, 0.9%), congenital myopathy (N = 1, 0.3%) and spinal muscular atrophy (N = 2, 0.6%). Based on the diagnostic criteria proposed by Hagberg^11^, it was found that they represented the following types of cerebral palsy: spastic—93.3% (N = 223), mixed—5% (N = 12) and ataxic—1.7% (N = 4). No cases of dyskinetic type were found. In the group with spastic cerebral palsy, tetraplegia was found in 76 children (34.1%), diplegia in 90 children (40.4%) and hemiplegia in 57 children (25.6%). Principal diagnoses were found to co-occur with the following additional diagnoses: hypothyroidism 4.3% (N = 14) and symptomatic epilepsy 26.3% (N = 86)—the patients with these diagnoses were receiving anti-epileptic drugs and thyroid hormone supplementation, respectively. In the group, 8% of the children had received operative treatment for hydrocephalus (condition after operative treatment for hydrocephalus) and 3 individuals (0.9%)—had hydrocephalus, but it had not been treated surgically (two children after a surgery for myelomeningocele (sasMMC) and in one child with isolated hydrocephalus).

Selected numerical characteristics were shown for z-score Ht, z-score Wt, z-score HC, z-score BMI as well as z-score HCI, such as arithmetic mean ($\overline{x}$), median (Me), standard deviation (*s*), minimum value (Min), maximum value (Max), 25th centile (*c*_25_) and 75th centile (*c*_75_). In previous analyses and publications, z-score Ht^19^, z-score HC^20^ and z-score HCI^20^ were already used and presented. The mean and median for z-score Ht in the study group assumed values lower than − 1 and higher than − 2. On the other hand, the mean and median for z-score Wt, z-score HC and z-score BMI assumed values lower than zero and higher than − 1. The mean and median values for z-score HCI were higher than zero and lower than 1 (Table 1D).

No cases met the criteria for proportional micro-SBS (N = 0) or proportional macro-SBS (N = 0), for which indicators of proportion, i.e. BMI and HCI were to assume normal values. Due to this, further in the article the term micro-SBS will refer to disproportional micro-SBS, while macro-SBS will refer to disproportional macro-SBS. Micro-SBS was found in 7.3% cases (N = 24) and macro-SBS in 0.6% cases (N = 2) (Table 1E).

The statistical characteristics of the GMFCS score in the entire study group and in micro/macro-SBS are presented in the Table 1F. The mean GMFCS-scores are higher for micro-SBS than for macro-SBS. The statistical characteristics of the GMFCS score in the entire study group has been presented previously^20^.

Subsequent analyses examined correlations between existing development disorders, i.e. micro/macro-SBS and disorders/syndromes occurring with neurodysfunction (Table 2A), taking into account classification with regard to etiopathogenesis, presence and character of encephalopathy (Table 2B), classification with regard to presence and character of encephalopathy (Table 2C), types of cerebral palsy (Table 3A), type of spasticity in cerebral palsy (Table 3B), epilepsy (Table 3C), hypothyroidism (Table 3D), hydrocephalus treated surgically (Table 3E), as well as hydrocephalus not treated surgically (Table 3F). After statistically significant relationships were identified, the analyses also focused on the co-existence of type of spasticity in cerebral palsy and hydrocephalus not treated surgically (Table 3G). The correlations were then calculated between existing development disorders: micro/macro-SBS and GMFCS I–V in the entire group (Table 4A), GMFCS A–C in the entire group (Table 4B), GMFCS I–V in the CP subgroup (Table 4C), GMFCS A–C in the cerebral palsy subgroups (Table 4D).

**Table 2:** Developmental disorders of body structure and: principal diagnosis (A), classification of encephalopathy (B-C).

A. Units and syndromes running with neurodysfunction (principal diagnosis)	Developmental disorders of body structure (*p* = 0.172; Cp = 0.574)				In total
	Micro-SBS		Macro-SBS		
	N (%)	ASR	N (%)	ASR	N (%)
ACM	1 (100%)	0.3	0	− 0.3	1
HCP	0	− 3.5	1 (100%)	3.5	1
DS	2 (100%)	0.4	0	− 0.4	2
ES	1 (100%)	0.3	0	− 0.3	1
DGS	1 (100%)	0.3	0	− 0.3	1
FAS	1 (100%)	0.3	0	− 0.3	1
CP	15 (93.8%)	0.3	1 (6.3%)	− 0.3	16
HMSN	1 (100%)	0.3	0	− 0.3	1
AMC&N	1 (100%)	0.3	0	− 0.3	1
SMA	1 (100%)	0.3	0	− 0.3	26
In total	24 (92.3%)		2 (7.7%)		26 (100%)

B. Classification with regard to etiopathogenesis, presence and character of encephalopathy	Developmental disorders of body structure (p = 0.218; Cp = 0.426)				In total
	Micro-SBS		Macro-SBS		
	N (%)	ASR	N (%)	ASR	N (%)
NTDs	1 (50%)	− 2.3	1 (50.0%)	2.3	2
GD	4 (100%)	0.6	0	− 0.6	4
TE	1 (100%)	0.3	0	− 0.3	1
CP	15 (93.8%)	0.3	1 (6.3%)	− 0.3	16
NMD	3 (100%)	0.5	0	− 0.5	3
In total	24 (92.3%)		2 (7.7%)		26(100%)

C. Classification with regard to presence and character of encephalopathy	Developmental disorders of body structure (p = 0.595; Cp = 0.104)				In total
	Micro-SBS		Macro-SBS		
	N (%)	ASR	N (%)	ASR	N (%)
NPE	21 (91.3%)	− 0.5	2 (8.7%)	0.5	23
NMD	3 (100%)	0.5	0	− 0.5	3
In total	24 (92.3%)		2 (7.7%)		26 (100%)

* micro-SBS* microsomic body structure, *macro-SBS* macrosomic body structure, *ACM* Arnold-Chiari malformation, *HCP* isolated hydrocephalus, *DS* Down’s syndrome, *ES* Edwards syndrome, *DGS* DiGeorge syndrome, *FAS* fetal alcohol syndrome, *CP* cerebral palsy, *HMSN* hereditary motor and sensory polyneuropathy, *AMC&N* arthrogryposis multiplex congenita with neuropathy, *SMA* spinal muscular atrophy, *NTDs* neural tube defects, *GD* genetic disorders, *TE* toxic encephalopathy, *CP* cerebral palsy, *NMD* neuromuscular disorders, *NPE* non-progressive encephalopathy, *NMD* neuromuscular disorders, *N* numbers of patients, *%* percent, *p* probability value calculated by chi-square test of independence, Cp—Pearson’s Contingency Coefficient C ,Cp ≥ 0, values distant from 0 reflect a relationship; values approaching 1 correspond to a near-perfect association, ASR—Adjusted Standardized Residuals, values > 1.96 reflect a higher number, and those below < -1.96 correspond to a lower number than a random distribution.

**Table 3:** Developmental disorders of body structure and: type of CP (A), type of spasticity (CP) (B), epilepsy (C), hypothyroidism (D), hydrocephalus treated/not treated surgically (E/F), type of spasticity (CP) and hydrocephalus not treated surgically (G).

Types of CP	A. Developmental disorders of body structure				In total		
	Micro-SBS		Macro-SBS				
	N (%)	ASR	N (%)	ASR	N (%)		
Spastic type	15 (93.8%)	–	1 (6.3%)	–	16		
Atactic type	0	–	0	–	0		
Mixed type	0	–	0	–	0		
In total	15 (93.8%)		1 (6.3%)		16 (100%)		

Type of spasticity (CP)	B. Developmental disorders of body structure (p = 0.024; Cp = 0.564)				In total		
	Micro-SBS		Macro-SBS				
	N (%)	ASR	N (%)	ASR	N (%)		
Diplegia	5 (100%)	0.7	0	− 0.7	5		
Hemiplegia	1 (50%)	− 2.7	1 (50%)	2.7	2		
Tetraplegia	9 (100%)	1.2	0	− 1.2	9		
In total	15 (93.8%)		1 (6.3%)		16 (100%)		

Accompanying diagnosis Epilepsy	C. Developmental disorders of body structure (p = 0.727; Cp = 0.068)				In total		
	Micro-SBS		Macro-SBS				
	N (%)	ASR	N (%)	ASR	N (%)		
Present	9 (90.0%)	− 0.3	1 (10.0%)	0.3	10		
Lack	15 (93.8%)	0.3	1 (6.3%)	− 0.3	16		
In total	24 (92.3%)		2 (7.7%)		26 (100%)		

Accompanying diagnosis Hypothyroidism	D. Developmental disorders of body structure (p = 0.595; Cp = 0.104)				In total		
	Micro-SBS		Macro-SBS				
	N (%)	ASR	N (%)	ASR	N (%)		
Present	3 (100%)	0.5	0	− 0,5	3		
Lack	21 (91.3%)	− 0.5	2 (8.7%)	0,5	23		
In total	24 (92.3%)		2 (7.7%)		26 (100%)		

Hydrocephalus treated surgically	E. Developmental disorders of body structure				In total		
	Micro-SBS		Macro-SBS				
	N (%)	ASR	N (%)	ASR	N (%)		
Lack	24 (92.3%)	–	2 (7.7%)	–	26		
Present	0	–	0	–	0		
In total	24 (92.3%)		2 (7.7%)		26 (100%)		

Hydrocephalus not treated surgically	F. Developmental disorders of body structure (p < 0.001; Cp = 0.569)				In total		
	Micro-SBS		Macro-SBS				
	N (%)	ASR	N (%)	ASR	N (%)		
Lack	24 (96%)	3.5	1 (4%)	− 3.5	25		
Present	0	− 3.5	1 (100%)	3.5	1		
In total	24 (92.3%)		2 (7.7%)		26 (100%)		

Hydrocephalus not treated surgically	G. Type of spasticity (CP)						In total
	Tetraplegia		Hemiplegia		Diplegia		
	N (%)	ASR	N (%)	ASR	N (%)	ASR	N (%)
Lack	76 (34.1%)	–	57 (25.6%)	–	90 (40.4%)	–	223
Present	0	–	0	–	0	–	0
In total	76 (34.1%)		57 (25.6%)		90 (40.4%)		223(100.0%)

*CP* cerebral palsy, *micro-SBS* microsomic body structure, *macro-SBS* macrosomic body structure, *N* numbers of patients, % percent, *p* probability value calculated by chi-square test of independence, *Cp* Pearson’s Contingency Coefficient C ,Cp ≥ 0, values distant from 0 reflect a relationship; values approaching 1 correspond to a near-perfect association, *ASR* Adjusted Standardized Residuals, values > 1.96 reflect a higher number, and those below < − 1.96 correspond to a lower number than a random distribution.

**Table 4:** Developmental disorders of body structure and: GMFCS I-V in the entire study group (A), GMFCS A-C in the entire study group (B), GMFCS I-V in the entire study group (C), GMFCS A-C in the entire study group (D).

A. Developmental disorders of body structure in the entire study group p = 0.558, Cp = 0.320							
GMFCS I–V	Micro-SBS			Macro-SBS		In Total	
	ASR	N (%)		ASR	N (%)		
GMFCS I	4 (80.0%)	− 1.1		1 (20%)	1.1	5	
GMFCS II	5 (83.3%)	− 0.9		1 (16.7%)	0.9	6	
GMFCS III	3 (100%)	0.5		0	− 0.5	3	
GMFCS IV	4 (100%)	0.6		0	− 0.6	4	
GMFCS V	8 (100%)	1		0	− 1	8	
In Total	24 (92.3%)		2 (7.7%)			26 (100%)	

B. Developmental disorders of body structure in the entire study group p = 0.228, Cp = 0.324							
GMFCS A-C	Micro-SBS			Macro-SBS		In Total	
	ASR	N (N%)		ASR	N (N%)		
GMFCS A	9 (81.8%)	− 1.7		2 (18.2%)	1.7	11	
GMFCS B	3 (100%)	0.5		0	− 0.5	3	
GMFCS C	12 (100%)	1.4		0	− 1.4	12	
In Total	24 (92.3%)		2 (7.7%)			26 (100%)	

C. Developmental disorders of body structure in the CP subgroup p = 0.113, Cp = 0.56							
GMFCS I-V	Micro-SBS			Macro-SBS		In Total	
	ASR	N (%)		ASR	N (%)		
GMFCS I	1 (50%)	− 2.7		1 (50%)	2.7	2	
GMFCS II	3 (100%)	0.5		0	− 0.5	3	
GMFCS III	2 (100%)	0.4		0	− 0.4	2	
GMFCS IV	3 (100%)	0.5		0	− 0.5	3	
GMFCS V	6 (100%)	0.8		0	− 0.8	6	
In Total	15 (93.8%)		1 (6.2%)			16 (100%)	

D. Developmental disorders of body structure in the CP subgroup p = 0.309, Cp = 0.36							
GMFCS A-C	Micro-SBS			Macro-SBS		In Total	
	ASR	N (N%)		ASR	N (N%)		
GMFCS A	4 (80%)	− 1.5		1 (20%)	1.5	5	
GMFCS B	2 (100%)	0.4		0	− 0.4	2	
GMFCS C	9 (100%)	1.2		0	− 1.2	9	
In total	15 (93.8%)		1 (6.2%)			16 (100%)	
*micro-SBS* microsomic body structure, *macro-SBS* macrosomic body structure, *CP* cerebral palsy, *GMFCS* Gross Motor Function Classification System, *N* numbers of patients, % percent, *p* probability value calculated by chi-square test of independence, *Cp* Pearson’s Contingency Coefficient C, *Cp ≥ 0*, values distant from 0 reflect a relationship; values approaching 1 correspond to a near-perfect association, *ASR* Adjusted Standardized Residuals, values > 1.96 reflect a higher number, and those below < − 1.96 correspond to a lower number than a random distribution							

E. The difference in the GMFCS I–V score between macro-SBS and micro-SBS U = 8.50, p = 0.154							
Parameter	Study group	N	$\overline{x}$	Me	*s*	Min	Max
GMFCS I–V	Micro-SBS	24	3.29	3.5	1.55	1	5
GMFCS I–V	Macro-SBS	2	1.50	1.5	0.71	1	2

F. The difference in the GMFCS I–V score between macro-SBS and micro-SBS U = 9.00, p = 0.185							
Parameter	Study group	N	$\overline{x}$	Me	*s*	Min	Max
GMFCS A–C	Micro-SBS	24	2.12	2.5	0.95	1	3
GMFCS A–C	Macro-SBS	2	1.00	1.0	0.00	1	1

*micro-SBS* microsomic body structure, *macro-SBS* macrosomic body structure, *U* value of the Mann–Whitney U test, *p* test probability index.

Micro-SBS was found in patients (Tables 2A–C, 3A,B) with:

- Non-progressive encephalopathy (N = 21): in 15 patients with cerebral palsy (exclusively with spastic type: five patients with diplegia, one with hemiplegia and nine with tetraplegia), in four patients with genetic disorders (two patients with Down’s syndrome, one patient with Edwards syndrome and one patient with DiGeorge syndrome), in one patient with neural tube defects (with Arnold-Chiari malformation) and in one patient with toxic encephalopathy (with fetal alcohol syndrome),
- Neuromuscular diseases (N = 3): in one patient with hereditary motor and sensory polyneuropathy, one patient with arthrogryposis multiplex congenita with neuropathy and one patient with spinal muscular atrophy.

Out of 76 patients with spastic type cerebral palsy, 15 presented as micro-SBS. Hence in the group of patients with spastic type cerebral palsy, micro-SBS occurred at a rate of 19.7%. Four out of 23 patients with genetic disorders were found with micro-SBS, i.e. micro-SBS occurred in patients with genetic disorders at a rate of 17.4%.

Micro-SBS was not identified in patients with progressive encephalopathy (Table 2C) or in those with hydrocephalus, subjected to operative treatment (Table 3E), or not subjected to such treatment (Table 3F).

Macro-SBS was found only in patients with non-progressive encephalopathy (N = 2): 1 patient with neural tube defects (with hydrocephalus, the patient was not subjected to surgery due to hydrocephalus) and one patient with cerebral palsy (spastic type: hemiplegia) (Tables 2C–E, 3A,B,F). Hemiplegia was not identified in children with hydrocephalus not treated surgically (Table 3G). Macro-SBS occurred in patients with spastic type cerebral palsy at a rate of 1.3% (1 patient in a group of 76).

The results show the following statistically significant relationships:

- Micro-SBS is rarely associated with hemiplegia (50%), macro-SBS frequently co-occurred with hemiplegia (50%); ASR in the former case amounts to − 2.7 and in the latter case to + 2.7 (p = 0.024) (Table 3B),
- Macro-SBS more frequently co-occurred with hydrocephalus not treated surgically in the study group of children (100%) (p < 0.001) (Table 3F).

In other cases, no statistical significance was achieved (Table 2A,B) or it was impossible to carry out statistical analyses in three cases due to the occurrence of constant values (Tables 2C, 3D–G). There were no statistically significant differences between micro-SBS and macro-SBS in the GMFCS I-V score and the GMFCS A-C score (p > 0.05) (Table 4E,F).

## Discussion

An individual’s development, on the one hand, is determined by his/her genotype, and on the other hand, it is modified by the impact of his/her environment^14^. It is believed that intrauterine hypotrophy and fetal macrosomia are associated with a wide range of pathological processes occurring during various stages of gestation^5^. In Poland this problem has been relatively overlooked. The scarce data published so far mainly relate to fetal macrosomia in infants born to mothers with gestational diabetes^52,53^. Low birth weight in neonates occurs at a rate of 3–10%^2,5^. The most recent reports suggest that macrosomia is more and more frequently identified in neonates worldwide^6^. The prevalence of fetal macrosomia in the general population is in the range of 6–10%^7,54^. On the other hand, central nervous system anomalies are estimated to occur at a rate of about 1.4–1.6 per 1000 live births^55^. The prevalence of disproportional micro/macro-SBS amounted to 7.3% and 0.6%, respectively, in the children and adolescents affected by syndromes or diseases associated with neurodysfunction. The current study showed that no cases met the criteria for proportional micro/macro-SBS (for which indicators of proportion, i.e. BMI and HCI were to assume normal values). This may be associated with impaired differentiation of body proportions in children with micro/macro-SBS. Hence, the authors decided to use the terms micro/macro-SBS without the adjectives proportional/disproportional.

Micro/macro-SBS was identified in only patients with spastic type cerebral palsy. Due to this it was impossible to carry out statistical analyses examining the co-occurrence of micro/macro-SBS and spastic types of cerebral palsy. Micro-SBS was found among the patients with spastic types of cerebral palsy at a rate of 19.7% and macro-SBS at a rate of 1.3%. A statistically significant relationship was found between micro/macro-SBS and type of spasticity (cerebral palsy). Micro-SBS rarely co-occurs with hemiplegia, while macro-SBS is frequently found to co-exist with hemiplegia. Yamada et al. investigated the risk of cerebral palsy linked to neonatal encephalopathy in children with a birth weight of 4.0 kg or higher. In a retrospective study the researchers examined information related to 132 singletons diagnosed with this type of cerebral palsy, and they analyzed the data by reference to the national statistics related to birthweight categories identified in Japanese infants. Their findings confirmed a greater risk of cerebral palsy linked to neonatal encephalopathy in Japanese infants presenting with macrosomia at birth^56^_._ Dahlseng et al., in a study involving Norwegian term-born singleton infants, investigated the relationship between cerebral palsy incidence and body weight and length as well as head circumference identified at birth. In their analyses the researchers applied standard deviation z-scores for the above size measures, as well as ponderal index at birth. Comparative analyses took into account data retrieved from the Cerebral Palsy Registry of Norway (398 children with cerebral palsy) and from the Medical Birth Registry of Norway (490,022 infants with normal development). The findings showed that a greater risk of cerebral palsy (particularly bilateral spastic cerebral palsy) corresponded to low body weight as well as high and low z-scores for head circumference and body length at birth. On the other hand, the incidence of unilateral spastic cerebral palsy was related only to low z-scores, while increased risk of spastic quadriplegic and dyskinetic cerebral palsy was identified in children with the largest head circumference and greatest body length, as well as low ponderal index^57^_._ The coexistence of micro-SBS with spastic type cerebral palsy confirmed the encephalization theory about the relationship between size of brain and size of body^58^.

Micro-SBS occurred in patients with genetic disorders at a rate of 17.4%. It co-occurred with Down’s syndrome, Edwards syndrome and DiGeorge syndrome. In Down’s syndrome we can observe two types of growth restriction, associated with pituitary gland or thyroid gland functions. In the former case the dimensions of the whole body are decreased, and in the latter case we can observe short stature and obesity^59^. In DiGeorge syndrome, impaired development of structures and functions of the oral cavity, pharynx, cranial nerves and brain stem leads to feeding and swallowing difficulties^60^, which may affect nutritional status and growth processes^61^. Edwards syndrome is associated with intrauterine hypotrophy and, during the post-natal period, with insufficient body weight and short stature^62,63^. Likewise, micro-SBS was found in the patient with fetal alcohol syndrome—alcohol is a toxic substance inducing symmetric intrauterine hypotrophy^2^. Micro-SBS was also identified in the patient with neural tube defects—with Arnold-Chiari malformation. In another study, carried out in a group of children receiving operative treatment due to meningomyelocele (the study group) and healthy peers (control group) it was shown that the length of the lower limb in the children from the study group develops at a lower kinetic level, compared to the controls. The mean annual increase in this characteristic is lower in the children in the study group compared to the controls, which is linked with short stature; however, the children in the study group present a tendency to accumulate fatty tissue^64^, which is not typical for micro-SBS. Additionally, isolated cases of micro-SBS were found in patients with damaged lower motor neurons in neuromuscular diseases: with hereditary motor and sensory polyneuropathy, arthrogryposis multiplex congenita with neuropathy or spinal muscular atrophy.

The current study also found a statistically significant relationship that shows that macro-SBS co-occurs more frequently with hydrocephalus not treated surgically. A co-occurrence of hydrocephalus and macrosomia was also reported by Muller et al. in patients with basal cell nevus syndrome, also known as Gorlin syndrome^65^. Macrosomia in these individuals has been hypothesized to result from de novo deletions of the paternal allele specifically, possibly due to the loss of one or more as of yet unidentified imprinted genes^66,67^. Muller et al. characterize new phenotypic features not consistent with basal cell nevus syndrome—metopic craniosynostosis, obstructive hydrocephalus, macrosomia and developmental delay^65^. No statistically significant differences were found between GMFCS and micro-SBS and macro-SBS.

Another article reported that children and adolescents with neurodysfunction present growth defects. Short stature in children and adolescents with neurodysfunction co-occurs with hypothyroidism over the whole group studied, tetraplegia in the subgroup with spastic type cerebral palsy and in patients receiving operative treatment due to myelomeningocele with hydrocephalus belonging to the group of neural tube defects^19^. There is also a relationship between the coexistence of absolute microcephaly (z-score HC < 2 standard deviations, z-score HCI < 2 standard deviations) and epilepsy in this group^20^.

It seems that the hypothesis stating that the population of children and adolescents includes individuals with body dimensions (body weight, body height, head circumference) below and above the norm has been confirmed. Some of the children and adolescents with syndromes or diseases associated with neurodysfunction presented micro/macro-SBS (the former being far more common). Statistically significant relationships were found between co-occurring micro/macro-SBS and type of spasticity (cerebral palsy) as well as hydrocephalus not treated surgically. Head MRI is important in assessing the extent of brain changes in children with cerebral palsy^68^. Finally, it is worth adding that each of the disorders of growth and body proportions should also be included in a differential diagnosis taking into account endocrine, metabolic and genetic diseases^69^.

## Limitation

The research was retrospective in nature and there were relatively insufficient data from medical records. However, from a clinical point of view, it should be emphasized that there are also other factors that significantly affect the course of developmental disorders. For example, in future prospective studies more attention should be paid to hormonal balance and head imaging studies, as well as extended interview data.

The following sources of potential bias were identified in the methodology of the study:

1. The high exclusion rate, amounting to 88%, may adversely affect representativeness of the sample with respect to the cohort studied. Hospital readmissions were excluded in order to eliminate the data from the same patient, as a result of which a given patient was assessed only once. However, in further research it would be worthwhile to consider handling participants with multiple admissions as separate cases, or using the mean z-score for their measures, thereby reducing a source of bias. The study did not take into account patients without an identified diagnosis—the inclusion/exclusion criterion applied was defined as presence/absence of a defined diagnosis. The potential source of bias is linked with the fact that it is impossible to determine unequivocally whether or not micro-SBS, or macro–SBS occurred in this group of patients. Hospitalisations were also disregarded in the case of patients who presented combinations of congenital disorders of the nervous system or neurological syndromes (e.g. Down’s syndrome, neural tube defect or phenylketonuria co-occurring with cerebral palsy). The potential source of bias is the fact that the distinction between congenital disorders and neurological syndromes is arbitrary, as i) both represent congenital conditions, and ii) the distinction may be an artifact of different nosologies (e.g. etiological diagnosis such as trisomy 21, and descriptive diagnosis such as neural tube defect) although they are more likely to co-occur than not (e.g. neural tube defects are more common in patients with genetic syndromes than those without). Our study did not take into account patients with neural tube defects co-existing with Down syndrome, or another syndrome resulting from another chromosome aberration or from a single gene mutation. Three patients excluded from the study presented with the following co-existing conditions: Down syndrome and cerebral palsy (spastic hemiplegia, condition following cardiogenic stroke in the first six months of life), meningomyelocele and cerebral palsy (spastic diplegia, premature birth complications, periventricular leukomalacia), phenylketonuria and cerebral palsy (spastic diplegia, premature birth complications, periventricular leukomalacia).
2. Unknown bias related to incomplete data in the medical records (e.g. if some patients are less likely to be measured in some regard. For example, height and weight may not be measured as frequently in patients with tetraplegic type of cerebral palsy and with myelomeningocele because the operation is cumbersome).
3. Bias related to patient’s age at admission (in our facility pre-school and older school age children are more frequently present in the inpatient unit compared to early school age children. Notably, the rate of growth differs during the above specific stages of development: pre-school age versus early/primary school age versus older/secondary school age, associated with puberty—pubertal spurt in body height preceding adolescence).

### Clinical implications

1. In undiagnosed children with neurodysfunction from birth and micro/macro-SBS, an interview should be conducted for risk factors for perinatal brain injury and fetal alcohol exposure.
2. Undiagnosed children with micro/macro-SBS and neurodysfunction admitted to the Department of Neurological Rehabilitation should receive a referral to:
  - the Outpatient Clinic/Department of Neurology to: perform an MRI of the brain and spinal cord to confirm/exclude cerebral palsy and neural tube defect; perform ENG and EMG to confirm/exclude neuromuscular diseases,
  - the Genetic Outpatient Clinic in order to perform diagnostics to confirm/exclude genetic and neuromuscular diseases.
3. Children with micro/macro-SBS require an in-depth assessment of their nutritional status based on BMI and, if indicated, adequate therapeutic management.
4. The prognosis of motor development, as assessed by GMFCS, cannot be related to micro-SBS and macro-SBS in children with established diagnosis and neurodysfunction. The claim that gross motor development will be worse (higher GMFCS score) in micro-SBS than in macro-SBS is not supported by statistically significant results.

## Conclusions

1. Patients with micro-SBS and macro-SBS structure present abnormal differences in body proportions.
2. Micro-SBS and macro-SBS were not identified in patients with progressive encephalopathy.
3. Micro-SBS was most frequently identified in patients with spastic CP. Hemiplegia is rarely accompanied with micro-SBS and frequently with macro-SBS.
4. Patients receiving treatment for hydrocephaly did not present developmental defects reflected by micro/macro-SBS.
5. Macro-SBS co-occurred with hydrocephaly not treated surgically.
